# Works in Preparation for the New Sydenham Society

**Published:** 1861-03

**Authors:** Richard J. Dunglison

**Affiliations:** Hon. Local Secretary, Philada.


WORKS IN PREPARATION FOR THE NEW SYDENHAM SOCIETY.—In addition to works already published, the following are in course of preparation for the Society:—

Vogel and Neubauer on the Examination of the Urine: a Manual intended for the Assistance of the Practical Physician. Translated by Dr. Markham. With Lithographs and Wood-cuts.

A Year-book of Medicine and Surgery for 1860.

Frerichs's Clinical Account of Diseases of the Liver, vol. ii. Translated by Dr. Murchison. With Wood-cuts.

Casper's Manual of Forensic Medicine. Translated by Dr. G. Balfour, of Edinburgh.

Czermak's Monograph on the Laryngoscope and its Practical Applications. Translated by Dr. G. D. Gibb. With Wood-cuts.

Professor Donders on the Diseases of Accommodation of the Eye. With Notes by the Author. Translated by Dr. W. D. Moore, of Dublin.

Smellie's Midwifery. Reprinted, with Notes and Preface, etc., bringing the Work up to the present Standard of Knowledge. By Professor Simpson, of Edinburgh.

RICHARD J. DUNGLISON,
*Hon. Local Secretary, Philada.*

